# Clinical efficacy of different androgen deprivation therapies for prostate cancer and evaluation based on dynamic-contrast enhanced magnetic resonance imaging

**DOI:** 10.3389/abp.2024.12473

**Published:** 2024-05-15

**Authors:** WenXiao Guo, MengZhu Li

**Affiliations:** Department of Radiology, Wuhan Fourth Hospital, Wuhan, Hubei, China

**Keywords:** prostate cancer, clinical effect, DCE-MRI, intermittent androgen deprivation therapy, continuous androgen deprivation therapy

## Abstract

**Objective:**

To evaluate the clinical efficacy of different androgen deprivation therapies for prostate cancer (PCa) based on dynamic-contrast enhanced magnetic resonance imaging (DCE-MRI).

**Methods:**

104 patients with PCa were studied, all of whom were treated with androgen deprivation therapy. The patients were divided into a continuous group (continuous androgen deprivation therapy) and an intermittent group (intermittent androgen deprivation therapy) by random number table method, 52 cases/group. The therapeutic effect and DCE-MRI indices were compared and the relationship between DCE-MRI indices and clinical efficacy and the evaluation value of therapeutic efficacy were analyzed.

**Results:**

The objective response rate (ORR) of the intermittent group was higher than that of the continuous group (*p* < 0.05), and there was no significant difference in disease control rate (DCR) between the two groups (*p* > 0.05). After treatment, volume transfer coefficient (K^trans^), reverse transfer constant (K_ep_), volume fraction (Ve), blood volume (BV), and blood flow (BF) in both groups were lowered, and those in the intermittent group were lower than the continuous group (*p* < 0.05). K^trans^, K_ep_, Ve, BF, and BV in the ORR group were lower than those in the non-ORR group (*p* < 0.05). K^trans^, K_ep_, Ve, BF, and BV were correlated with the therapeutic effect of PCa (*p* < 0.05). The AUC value of the combined detection of DCE-MRI indices in evaluating the therapeutic effect of PCa was greater than that of each index alone (*p* < 0.05).

**Conclusion:**

Compared with continuous androgen deprivation therapy, intermittent androgen deprivation therapy has better clinical efficacy in the treatment of PCa, and DCE-MRI indices are related to the treatment efficacy of PCa and have an evaluation value.

## Introduction

Prostate cancer (PCa) mainly refers to the pathological changes in the tissues around the prostate gland affected by various complex factors, resulting in the growth dysfunction or irregular growth of acinar cells and eventually inducing malignant tumors (Mori et al., 2022). Androgen deprivation therapy (ADT) is the main method to treat PCa, which can block androgens from testis and adrenal gland and has become the gold standard in the treatment of advanced PCa. However, total ADT can lead to adverse reactions in patients, such as hot flashes, gynecomastia, and osteoporosis, which significantly affect the quality of life of patients (Kishan et al., 2022). In recent years, intermittent androgen deprivation has become increasingly prominent in prolonging androgen resistance and reducing adverse reactions and has gradually attracted attention in the treatment of PCa (Denmeade et al., 2022). Currently, it is believed that the premise and basis of tumor formation are tumor neovascularization, which is an important index to evaluate tumor growth, metastasis, and malignancy degree, so the evaluation of tumor hemodynamics is extremely essential (Qu et al., 2021). Dynamic-contrast enhanced magnetic resonance imaging (DCE-MRI) calculates volume transfer coefficient (K^trans^), reverse transfer constant (K_ep_), and extracellular volume fraction (Ve) values through the pharmacokinetic model, which can reflect the changes of neovascularization density and blood perfusion in tumor tissues physiologically and pathologically before the changes of morphology and thereby provide quantitative data on the therapeutic effect of tumors at the molecular level (Ghoreifi et al., 2023). This study evaluated the clinical efficacy of different androgen deprivation therapies in the treatment of PCa from the perspective of DCE-MRI and provided references for the evaluation of therapeutic efficacy of this disease.

## Materials and methods

### Clinical data

104 patients with PCa from January 2020 to January 2023 were studied, all of whom were treated with ADT. The patients were divided into a continuous group (continuous ADT) and an intermittent group (intermittent ADT) by random number table method, 52 cases/group. Study procedures were reviewed and approved by the Ethics Committee of Wuhan Fourth Hospital (IRB number: 201912WH10; Registration number: ChiCTR1900075593; Date Granted: 2019-12), and all study subjects gave informed consent and signed written consent.

Inclusion criteria: (1) Patients confirmed as PCa by transrectal ultrasound-guided puncture and patients staged at T3∼T4; ② Patients with expected survival greater than 6 months; ③ Patients with complete clinical data; ④ Patients with well tolerance to MRI.

Exclusion criteria: ① Patients with poor image quality; ② Patients with other prostate diseases; ③ Patients with other malignant tumor diseases; ④ Patients with a history of radiation therapy and endocrine therapy before enrollment; ⑤ Patients with a recent history of prostatic puncture (within 1 month before enrollment).

### Treatment methods

Intermittent group received intermittent ADT, that is, anti-androgen combined with medical castration. All patients took 50 mg bicalutamide tablets orally once daily (CORDEN PHARMA GMBH, H20140720). Two weeks later, 3.6 mg goserelin acetate sustained-release depot (AstraZeneca UK Limited, J20100126) was injected subcutaneously into the lower abdomen. Serum rostate specific antigen (PSA), testosterone, and DCE-MRI were measured monthly during the treatment. Treatment was given every 4 weeks until PSA dropped to the lowest level (4 ng/mL), after which treatment was stopped at 3 months from PSA stabilization, entering the treatment intermission. Subsequently, serum PSA and blood testosterone levels were measured monthly, and DCE-MRI examinations were conducted to determine lesion expansion and the emergence of new metastatic lesions. If serum PSA rose again to more than 10 ng/mL or 50% of the initial diagnosis, the second cycle of treatment was required until serum PSA reduced to the lowest value. When intermittent therapy was completed, the treatment was stopped until new metastatic lesions appeared or primary lesions expanded. All patients were treated for 4–6 months and followed up for more than 6 months.

Serum PSA is a specific marker of PCa according to Prostate Cancer Diagnosis and Treatment Guidelines in 2014. PSA levels less than 4.0 ng/mL are considered normal, while PSA levels greater than 10 ng/mL are considered high-risk PCa. In this study, serum PSA and testosterone were monitored with regular follow-up, and Imaging examinations were performed to see if any new lesions had developed or if existing lesions had expanded. Medication can be stopped when serum PSA is less than 4.0 ng/mL and serum testosterone is less than 20.0 ng/dL.

The continuous group was given continuous ADT after bilateral testicle resection. Patients were given 50 mg bicalutamide orally, qd. Patients with hormone resistance received 250 mg flutamide (TASLY Diyi, H19990144) tid rather than bicalutamide tablets. Serum PSA, testosterone, and DCE-MRI examinations were monitored monthly (Yu et al., 2010; Mottet et al., 2012). Serum PSA levels must be reduced to 4 ng/mL, and serum testosterone to 20 ng/dL.

Resistance was defined as serum testosterone <50 ng/dL with a continuous PSA increase greater than 10 ng/mL 1 week apart.

### DCE-MRI detection method

Siemens 1.5T MR Scanner was used for DCE-MRI examination with 8 channel array coil and Siemens Syngo.via equipment. The scanning sequence mainly included T_2_WI, T_1_WI, diffusion weighted imaging (DWI), and DCE-MRI. DCE-MRI sequence took FLASH 3D-VIBE, T_1_WI axis position, layer thickness 3.5 mm, TR 4.2 ms, TE 1.58 ms, FOV 260 mm × 260 mm, NEX1, inverse angle 12°, voxel size 1.4 mm × 1.4 mm × 3.5 mm. Gd-diethylenetriamine pentaacetic acid (Gd-DTPA) and 20 mL normal saline were prepared according to the standard of 0.2 mmol/kg, and Gd-DTPA and 20 mL normal saline were simultaneously injected with a high-pressure syringe at 2.5 mL/s starting from the fourth stage. The scan was repeated for 36 stages, with a total scanning time of 4.1 min. 20 images were obtained in each stage, and a total of 720 original images were obtained. The original DCE-MRI images were transmitted to SiemensSyngo.via, Tissue 4d was specified as the workflow, and quantitative parameter values were obtained through motion correction, alignment, processing, and film reading. Alignment and motion correction were performed on 720 original images. In the processing program, The region of interest of the whole prostate was manually sketched through axial, coronal, and sagittal views, K^trans^, K_ep,_ and Ve were quantified and measured twice to get an average value. The perfusion parameters blood volume (BV) and blood flow (BF) were calculated according to the first-pass effect of the drug. DCE-MRI were measured monthly during the treatment and follow-up in both the intermittent group and continuous group.

### Clinical efficacy evaluation

Efficacy evaluation was conducted according to the RECIST criteria (Uemura et al., 2022). Complete response (CR): all target lesions disappeared completely except nodular diseases. These target nodules should be evaluated and reduced to normal size (short diameter <10 mm). Partial response (PR): The total diameter of all target lesions is ≥30% below baseline. Target nodules are measured by short diameter to calculate total diameter, whereas all other target lesions are measured by longest diameter. Progressive disease (PD): The minimum sum of the target lesion diameters was used as reference, and the relative increase in the diameter sum was at least 20% (if the baseline measurement was minimal, the baseline was used as reference); In addition, an absolute increase of at least 5 mm in the diameter sum must be satisfied (the presence of one or more new lesions is also considered disease progression). Stable disease (SD): The reduction of target lesions did not reach PR, and the increase did not reach PD, the minimum sum of diameters can be used as a reference. Objective response rate (ORR): the proportion of patients with a complete response or partial response to treatment. Disease control rate (DCR): the percentage of patients with advanced cancer whose therapeutic intervention has led to a complete response, partial response, or stable disease. The comparison results are the data after the end of treatment compared with those before treatment, no data during follow-up were included.

### Observation measures

(1) Clinical efficacy and DCE-MRI indicators. (2) DCE-MRI indices were compared after treatment, and the influence of DCE-MRI indices on the therapeutic effect of PCa was analyzed by logistic regression, and the evaluation value of DCE-MRI on the therapeutic effect of ADT was analyzed by operator characteristic (ROC) curve.

### Statistical analysis

Statistical data were analyzed using SPSS 22.0 software, and differences in enumeration data (percentage) were compared using the χ^2^ test. Measurement data were expressed by ( $\bar{x}$ ± s) after normal test, and the differences were compared by *t*-test. DCE-MRI was evaluated for its efficacy in the treatment of PCa using logistic regression, and its effectiveness in ADT was evaluated using ROC curves. A statistically significant difference was indicated by *p* < 0.05.

## Results

### Clinical data

Clinical data from both groups were not significantly different. There was no significant difference in age, BMI, fasting blood glucose, blood lipid levels and PSA between the two groups. In addition, tumor related data, such as maximum tumor diameter, IPSS score and TNM staging, were not statistically significant (*p* > 0.05, Table 1).

**TABLE 1:** Comparison of clinical data between the two groups.

Items	Continuous group (*n* = 52)	Intermittent group (*n* = 52)	χ2/t	P
Age (years)	69.03 ± 5.37	70.15 ± 5.31	1.069	0.287
BMI (kg/m2)	22.74 ± 2.19	22.58 ± 1.97	0.392	0.696
FPG (mmol/L)	5.26 ± 1.03	5.19 ± 1.16	0.325	0.746
TG (mmol/L)	1.47 ± 0.28	1.51 ± 0.24	0.782	0.436
TC (mmol/L)	4.53 ± 0.81	4.60 ± 0.85	0.43	0.668
PSA (μg/L)	62.76 ± 3.56	63.01 ± 3.78	0.347	0.729
Maximum tumor diameter (cm)	7.95 ± 1.16	8.05 ± 1.24	0.425	0.672
IPSS score	24.15 ± 2.09	23.98 ± 2.76	0.354	0.724
TNM staging			0.158	0.691
T3	31	29		
T4	21	23		

Notes: BMI, body mass index; FPG, fasting plasma glucose; TG, triglyceride; TC, cholesterol; PSA, rostate specific antigen; IPSS, score, International Prostatism Symptom score.

### Clinical effect

The ORR of the intermittent group was higher than that of the continuous group (*p* < 0.05). Although there was no significant difference in DCR between the two groups (*p* > 0.05, Table 2), compared with the continuous group, the DCR in the intermittent group still showed an upward trend.

**TABLE 2:** Comparison of clinical efficacy between the two groups (n, %).

Groups	n	CR	PR	SD	PD	ORR	DCR
Continuous group	52	9	19	20	4	53.85	92.31
Intermittent group	52	13	25	11	3	73.08	94.23
*χ* ^2^						4.147	1.182
*P*						0.042	0.277

Notes: CR, complete response; PR, partial response; SD, standard deviation; PD, progressive disease; ORR, objective response rate; DCR, disease control rate.

### DCE-MRI indices of patients with different therapeutic effects

**FIGURE 1:**
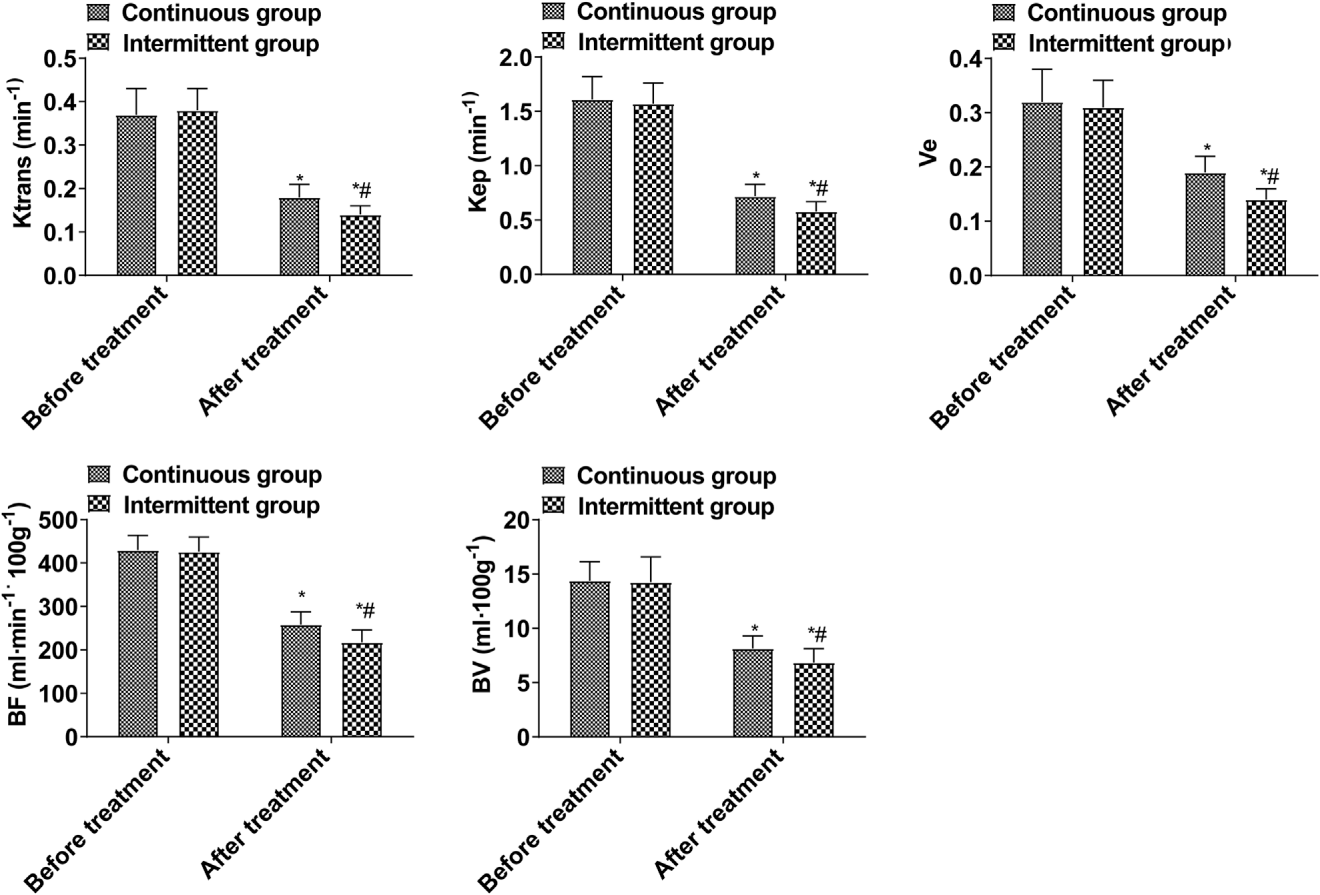
DCE-MRI indices (Compared with the group before treatment, *p < 0.05; Compared with the continuous group, #*p* < 0.05.).

**FIGURE 2:**
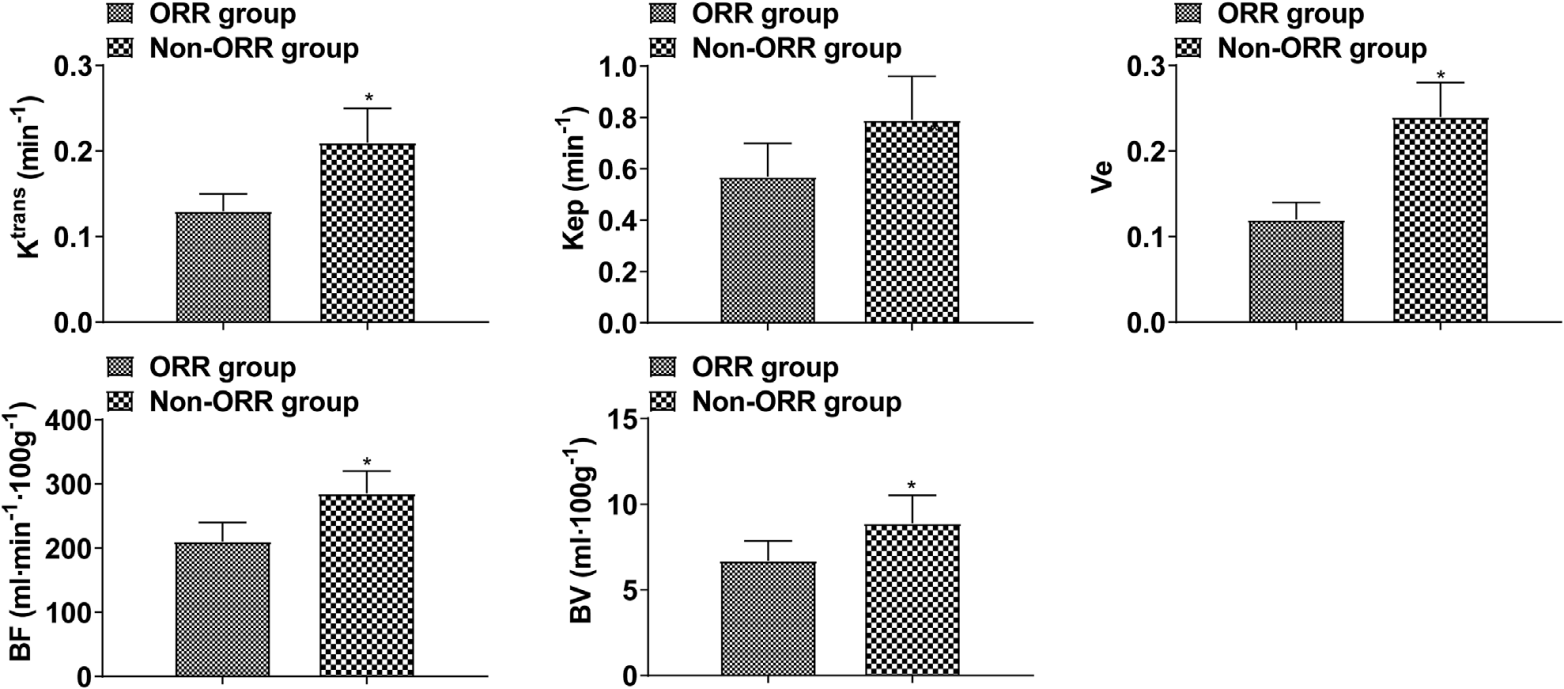
DCE-MRI indices in patients with different therapeutic effects (Compared with ORR group, *p < 0.05.).

As a result of treatment, K^trans^, K_ep_, Ve, BF, and BV decreased, and those decreased in the intermittent group significantly more than those in the continuous group (*p* < 0.05, Figure 1). These data show that the efficacy of intermittent ADT treatment is significantly higher than that of continuous ADT treatment group in terms of MRI performance. K^trans^, K_ep_, Ve, BF, and BV in the ORR group were lower than those in the non-ORR group (*p* < 0.05, Figure 2).

### Comparison of DCE-MRI indices of the therapeutic effect of PCa in the two treatment

Logistic regression analysis showed that K^trans^, K_ep_, Ve, BF, and BV values were correlated with the therapeutic effect of PCa (*p* < 0.05, Table 3). These suggests that these DCE-MRI parameters can well predict the clinical treatment efficacy of PCa.

**TABLE 3:** Analysis of the influence of DCE-MRI indexes on the therapeutic effect of prostate cancer.

Indices	*β*	*SE*	wald χ2	*OR*	95%*CI*	*P*
Ktrans	−5.43	1.477	13.516	0.004	<0.001–0.079	<0.001
Kep	−0.606	0.212	8.171	0.546	0.360–0.827	0.004
Ve	−3.969	1.516	6.854	0.019	0.001–0.369	0.009
BF	−0.025	0.013	3.698	0.975	0.951–1.000	0.055
BV	−0.153	0.25	0.375	0.858	0.526–1.401	0.541

Notes: K^trans^, volume transfer coefficient; K_ep_, reverse transfer constant; Ve, volume fraction; BF, blood flow; BV, blood volume; CI, confidence interval.

### Evaluation value of DCE-MRI indices on the therapeutic effect of PCa

**FIGURE 3:**
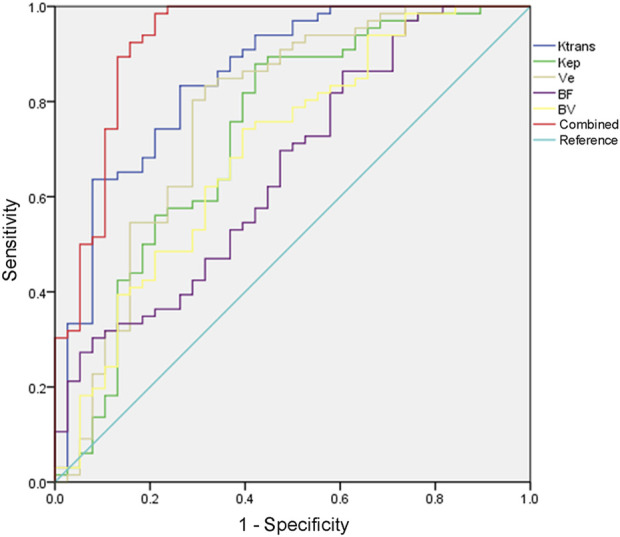
ROC curve of DCE-MRI to evaluate the therapeutic effect of PCa.

AUC value evaluated by the combined detection of K^trans^, K_ep_, Ve, BF, and BV was greater than that by each index alone (*p* < 0.05, Table 4; Figure 3). The combination of five items of DCE-MRI had the best predictive effect on PCa disease.

**TABLE 4:** Value analysis of DCE-MRI index in evaluating the therapeutic effect of prostate cancer.

Indices	Cut-off value	AUC	SE	*P*	95%CI
K^trans^	0.17 min^−1^	0.851*	0.041	<0.001	0.771–0.931
K_ep_	0.63 min^-1^	0.731*	0.055	<0.001	0.622–0.839
Ve	0.19	0.770*	0.053	<0.001	0.666–0.874
BF	253.86 mL min^−1^·100 g^−1^	0.658*	0.056	0.008	0.549–0.767
BV	8.01 mL·100g^−1^	0.698*	0.055	0.001	0.590–0.806
Combined	—	0.924	0.031	<0.001	0.863–0.986

Note: BV, blood volume; BF, blood flow; CI, confidence interval. Compared with combination, *p < 0.05.

## Discussion

PCa is a common malignant tumor of the male reproductive system. For patients with advanced PCa and those who cannot tolerate surgery, ADT is the most widely used therapeutic means at present (Cucchiara et al., 2019; Cipollari et al., 2022). ADT can inhibit the synthesis of vascular endothelial growth factor, induce endothelial cell apoptosis, inhibit the growth of androgen-dependent PCa tumors, and manage lymph node and bone metastasis (Al Salmi et al., 2020). At present, ADT is not only used for palliative treatment of advanced PCa but also adjuvant therapy before or after radical resection or radical radiotherapy to improve prognosis (Cereser et al., 2022). Continuous ADT can block androgens from testis and adrenal glands to the greatest extent, while intermittent ADT has become increasingly prominent in prolonging androgen resistance and reducing adverse reactions (Schaudinn et al., 2019; Carvajal et al., 2021). This study showed that the ORR of the intermittent group was higher than that of the continuous group, indicating that the clinical efficacy of intermittent ADT was better than that of continuous ADT. This was mainly because intermittent ADT could not prevent the transformation of androgen-dependent tumors into non-androgen-dependent tumors, but it could delay the transformation process and thus improve the therapeutic efficacy.

ADT is mainly recommended for symptomatic patients, patients with metastatic (M1) prostate cancer, or PSA dynamics characterized by rapid PSA rise or short PSA doubling time (less than 6 months). Intermittent ADT has been used as a method of monotherapy for ADT, which limits the incidence by periodically restoring serum testosterone levels. However, the clinical efficacy of intermittent ADT and continuous ADT is still controversial (Perera et al., 2020). The best method for ADT has not been clearly established. Traditionally, ADT is administered in a continuous manner, and the castration level of testosterone is ensured by repeated injections. This scheme usually continue to be used (Ravi and Choudhury, 2023). However, continuous use of GnRH analogues for ADT treatment can lead to considerable incidence of sexual and endocrine dysfunction and poor quality of life (QOL) outcomes. These problems led to the proposed intermittent application of ADT. The existing data of patients with locally advanced or recurrent non metastatic prostate cancer suggest that intermittent ADT treatment does not significantly affect OS outcome. Studies have shown that intermittent treatment may be the most beneficial for men with locally advanced or recurrent non metastatic prostate cancer and low baseline risk (PSA ≤1 ng/mL) (Bianchi et al., 2021).

ADT-treated PCa progresses to castration-resistant PCa after remission (Zhang et al., 2020). Patients may develop castration-resistant PCa within 5 years of treatment. Therefore, monitoring the efficacy of PCa after treatment is particularly considerable (Christophe et al., 2020). PSA is an index for monitoring the efficacy of PCa after radical surgery or conservative treatment. Although *post hoc* analyses of contemporary ARPI trials suggest that the depth of the initial PSA response is associated with favorable long-term outcomes. However, it could not reflect the changes of tumor morphology and microcirculation characteristics. PSA levels cannot truly reflect the histological and biological changes of tumors after endocrine therapy or radiation therapy, especially in cases with metastasis (Pesapane et al., 2020). ADT monitoring for PCa should therefore be applied with other indicators. Currently, it is believed that the growth and invasion of PCa depend on the tumor neovascularization. As the neovascularization wall fails to develop correctly, permeability increases, and blood is exchanged more rapidly between tumor and blood vessel, resulting in tumor invasion. MRI is the best imaging method for PCa, among which DCE-MRI quantitative analysis has been proven to be useful for evaluating tumor hemodynamics. Based on the difference in microvascular characteristics of lesions with different properties, DCE-MRI technology uses the difference in the time and concentration of contrast agent to reach normal tissues and pathological tissues with different properties to form different intensification methods, which can evaluate blood perfusion status of different tissues, so as to achieve the purpose of lesion diagnosis (Tamada et al., 2021; Forster et al., 2022).

DCE-MRI, combined with corresponding pharmacokinetic models, can obtain parameters that reflect tissue perfusion and permeation characteristics and quantitatively analyze the physiological characteristics of pathological tissues, which can be applied not only to the clinical diagnosis but also to the evaluation of the efficacy of tumor radiotherapy and chemotherapy (Prendeville et al., 2018). The common penetration parameters of DCE-MRI are K^trans^, Ve, and K_ep_. K^trans^ refers to the volume transport constant of contrast agent diffused from plasma into extravascular extracellular space, reflecting the level of vascular permeability in tissues. K_ep_ is the rate constant at which contrast agent diffuses from interstitial space to reflow into blood vessels. Ve refers to the volume ratio of EES per unit tissue volume. The perfusion parameters of DCE-MRI to reflect the blood perfusion status include BF and BV. BF represents the BF velocity in the blood vessels of the interest area per unit time, and BV represents the BV, both of which mainly reflect blood perfusion and depend on the microvascular density in the tissue (Arnoldner et al., 2022; Chatterjee et al., 2022; Fan et al., 2022; Nakayama et al., 2022). This study showed that DCE-MRI indices were all decreased after ADT, and K^trans^, K_ep_, Ve, BF, and BV in the ORR group were lower than those in the non-ORR group, suggesting that DCE-MRI indices may be used to evaluate the efficacy of ADT. This is mainly related to the reduction of PCa volume, gland atrophy, and fibrosis after ADT treatment (Lu et al., 2020). BF and BV of tumors after endocrine therapy are significantly reduced, and K^trans^ and K_ep_ are significantly reduced (Ikoma et al., 2020), which was consistent with the results of this study. ADT causes acinar atrophy, fibrosis, and basal cell hyperplasia in the prostate, resulting in a reduction in total glandular interstitial tissue and ultimately, total gland. In addition, after ADT, not only does vascular endothelial growth factor decrease with the decrease of androgen level, but also the BF of PCa tissues decreases, and cells show obvious apoptosis and necrosis, thus achieving anti-tumor angiogenesis, so K^trans^, K_ep,_ and Ve values are significantly reduced after endocrine therapy (Kim et al., 2021; Ueda et al., 2022). Further analysis found that K^trans^, K_ep_, Ve, BF, and BV were correlated with the therapeutic effect of PCa, and the AUC value of the combined detection of all indicators to evaluate the therapeutic effect of PCa was greater than 0.9, indicating that the DCE-MRI indices were correlated with the therapeutic effect of PCa.

In summary, As compared with continuous ADT, intermittent ADT improves PCa treatment outcomes, and DCE-MRI indicators are correlated with PCa treatment outcomes.

## Data Availability

The original contributions presented in the study are included in the article/Supplementary material, further inquiries can be directed to the corresponding author.
